# Built-in microscale electrostatic fields induced by anatase–rutile-phase transition in selective areas promote osteogenesis

**DOI:** 10.1038/am.2016.9

**Published:** 2016-03-04

**Authors:** Chengyun Ning, Peng Yu, Ye Zhu, Mengyu Yao, Xiaojing Zhu, Xiaolan Wang, Zefeng Lin, Weiping Li, Shuangying Wang, Guoxin Tan, Yu Zhang, Yingjun Wang, Chuanbin Mao

**Affiliations:** 1 School of Materials Science and Engineering, South China University of Technology, Guangzhou, China; 2 Department of Chemistry and Biochemistry, Stephenson Life Sciences Research Center, University of Oklahoma, Norman, OK, USA; 3 General Hospital of Guangzhou Military Command of PLA, Guangzhou, China; 4 Dental Department, Baoan Central Hospital of Shenzhen, Shenzhen, China; 5 Institute of Chemical Engineering and Light Industry, Guangdong University of Technology, Guangzhou, China; 6 School of Materials Science and Engineering, Zhejiang University, Hangzhou, Zhejiang, China

## Abstract

Bone has a built-in electric field because of the presence of piezoelectric collagen. To date, only externally applied electric fields have been used to direct cell behavior; however, these fields are not safe or practical for *in vivo* use. In this work, for the first time, we use a periodic microscale electric field (MEF) built into a titanium implant to induce osteogenesis. Such a MEF is generated by the periodic organization of a junction made of two parallel semiconducting TiO_2_ zones: anatase and rutile with lower and higher electron densities, respectively. The junctions were formed through anatase–rutile-phase transition in selective areas using laser irradiation on the implants. The *in vitro* and *in vivo* studies confirmed that the built-in MEF was an efficient electrical cue for inducing osteogenic differentiation in the absence of osteogenic supplements and promoted bone regeneration around the implants. Our work opens up a new avenue toward bone repair and regeneration using built-in MEF.

## INTRODUCTION

One of the major components of natural bone, collagen fibers, is a piezoelectric material.^1,2^ Therefore, during the constant motion of bone, bone cell activities and tissue formation take place in the presence of a built-in, not externally applied, electric field. Although electrical signals play an important role in wound healing and tissue regeneration,^3^ current studies have mainly used externally applied electric fields to direct cell behavior *in vitro*.^4–7^ When externally applied to cells, electrical signals are mainly transduced into intracellular signals through the cell membrane.^8,9^ The components of cell membranes, such as proteins, lipids and carbohydrates, are all charged molecules. When an external electric field is applied to cells, the charged components of the membranes are redistributed, and the voltage is hyperpolarized.^10–12^ These two processes were found to significantly affect cell behavior.

Inspired by these observations, various experiments have been carried out to use external electric fields to modulate cell differentiation on bone implants surface *in vitro*. Cui *et al.*^4^ used electric fields generated by special devices to modulate osteoblast differentiation on a polyelectrolyte multilayer film. They found the polyelectrolyte multilayer film coupled with an electrical stimulus could promote *in vitro* osteoblast differentiation in the early phase.^4^ When an electric field produced by a transformer-type device was applied to mesenchymal stem cells (MSCs) cultured on artificial extracellular matrices *in vitro*, it enhanced the osteogenic differentiation only in the presence of osteogenic supplements in the cell culture.^5^ In a separate study, conductive polymers were used as implant substrates to apply an electric stimulus to enhance the osteogenic differentiation of MSCs.^13,14^

**Figure 1:**
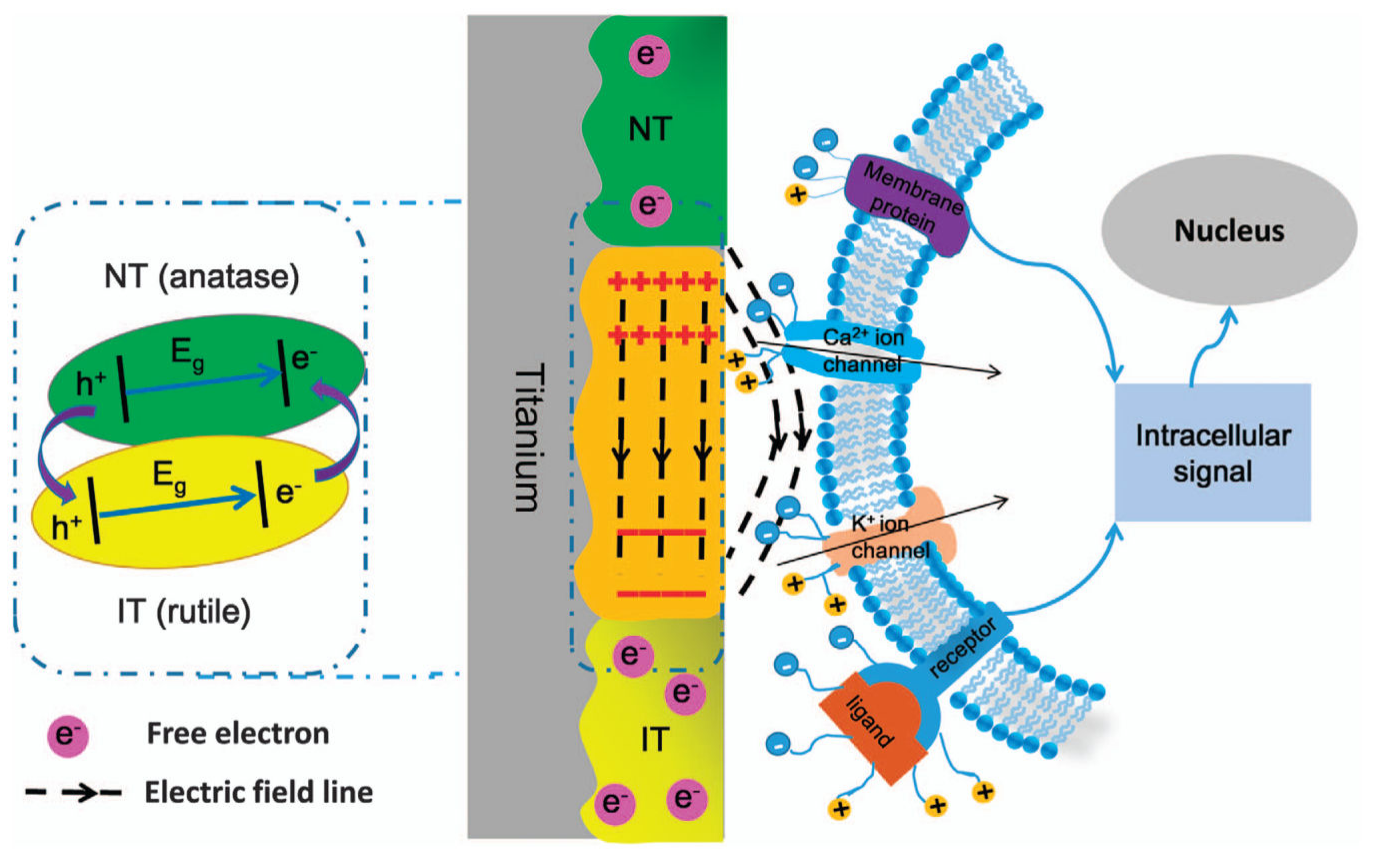
Illustration of the mechanism used to generate the microscale electrostatic field (MEF) and the interaction between the MEF and the stem cells. (Left) The MEF on the titanium surface. NT refers to the non-irradiated n-type semiconducting anatase TiO_2_ zone prepared by hydrothermal synthesis, and IT refers to the n-type semiconducting rutile TiO_2_ zone prepared by laser irradiation of the anatase TiO_2_ zone. The MEF was generated by the surface potential difference between the NT and IT zones. The diagram in the dashed line box illustrates the TiO_2_ phase junction of the NT and IT, as well as the electron transfer from the NT (rutile) to the IT (anatase) zone.^21,42^ (Right) Stem cell membrane with charged protein affected by MEF. As ion channels, membrane proteins, ligands and receptors are all charged with different surface potentials, their surface charges would be polarized under the guidance of MEF. The sustained built-in MEF enables the polarized stem cell surface species to transduce signals to the nucleus to activate osteogenic genes that results in enhanced osteogenesis.

Although electric fields have been used for stimulating cellular behaviors *in vitro*,^15–17^ currently, they are only applied externally to the implants through special devices that are not practical in eventual *in vivo* applications and can also pose potential safety issues when applied to the human body. Some components of bone, such as collagen fibers that are assembled into microscale fibrous patterns, are piezoelectric.^18^ Therefore, bone cells are actually living in a microscale electric environment.^2^ Inspired from this fact, we propose to generate a built-in microscale electric field (MEF) periodically distributed in titanium bone implants by periodically converting some anatase zones into rutile through periodic selective laser irradiation (Figure 1). Laser irradiation is an effective approach to converting anatase into rutile.^19,20^ During the laser scanning of a pristine anatase TiO_2_ surface film of a titanium implant, the irradiated TiO_2_ zone (IT) becomes an n-type semiconducting rutile phase with a greater electron density, whereas the nonirradiated parallel TiO_2_ zone (NT) remains an n-type semiconducting anatase phase with a lower electron density. Therefore, after laser scanning, the implant surface is made of the periodically organized parallel anatase and rutile zones with tunable intervals. The difference in the electron density between the parallel neighboring anatase and rutile zone results in the formation of an intrinsic built-in MEF (Figure 1). When anatase and rutile form a junction, the electrons flow from the anatase to the rutile phase and generate an electric field, making the surface potential of the rutile zone more negative than the anatase zone.^21,22^ Consequently, a periodically organized MEF is produced across the implant surface, as shown in Figure 1. This further drives us to study whether avoiding the use of an external electric field but using a built-in MEF can induce osteogenic differentiation and bone regeneration.

As ion channels, membrane proteins, ligands and receptors are all charged with different surface potentials, the surface charges of cells would be polarized under the guidance of a MEF.^6,10,23^ Because of the shielding effect of the plasma membrane, the macromolecules and ion channels embedded in the cell membrane would interact with an external electric field through Coulombic interactions.^10^ Under the sustained effect of a MEF, polarized stem cell surface proteins might transduce the electrical signal to the nucleus to activate osteogenic-related genes that might induce osteogenesis (Figure 1). Therefore, we hypothesized that a built-in MEF could induce osteogenic differentiation and bone regeneration.

## EXPERIMENTAL PROCEDURES

### Preparation of titanium substrates with built-in MEF

Biomedical titanium sheets with a thickness of 0.2 mm were obtained according to standard ASTM (American Society for Testing & Materials) F67-2000 from Baoji Qichen New Material Technology Co., Ltd. (Baoji, China). First, spontaneous TiO_2_ was prepared on the titanium substrates using a hydrothermal treatment process in a 5 mol l^−1^ NaOH (AR, Aladdin, Shanghai, China) solution at 80 °C for 12 h. Subsequently, the samples were placed in a 1 mmol l^−1^ HNO_3_ solution for 2 h and then heat treated at 550 °C for 2 h. Second, to fabricate the titanium substrate with a built-in MEF, an IPG solid-state laser (λ =1064 nm, Handslaser, Shenzhen, China) was used for selectively inducing phase transition at a laser power of 2.2 W and a laser scanning speed of 100 mm s^−1^. Meanwhile, with the help of CAD software (HL software, Handslaser), the intervals of the MEF could be finely tuned by changing the laser scanning pathways. For samples used for the animal experiments, cylindrical titanium rods (2 mm in diameter) were attached to a stepmotor to form the built-in MEF on their surface.

### MEF characterization

Scanning electron microscopy (ZEISS EVO18, Carl Zeiss Group, Oberkochen, Germany) coupled with energy-dispersive X-ray spectrum (INCA200, Oxford instruments, Oxford, UK) was used to characterize the morphology of the MEF. Kelvin probe force microscopy (Bruker Multimode 8, Santa Barbara, CA, USA) was used to construct the surface potential map of different parts of the MEF. The zeta potential of control 2 and control 3 was measured using a Zetameter (SurPASS, Anton Paar, Graz, Austria) by measuring the streaming potential in a KCl electrolyte (10^−3^ M). Mott–Schottky plots of the NT and IT samples were investigated in a conventional three-electrode cell using an electrochemical workstation (Zahner, Zennium, Kronach, Germany). The NT and IT with an electrode area of 2 cm^2^ were used as a working electrode. An Ag/AgCl electrode and a platinum electrode were used as the reference and counter electrode, respectively. Mott–Schottky plots were measured at a frequency of 1000 Hz in a phosphate buffer solution (Gibco, Carlsbad, CA, USA, 1 ×) at room temperature.

### Cell culture and seeding on the MEFs and controls

The MEFs and control samples were sterilized by immersion in 70% ethanol for 10 min, and then all sides of the samples were exposed to ultraviolet light for 15 min. Then, the samples were washed with sterilized phosphate buffer solution. Mouse bone marrow-derived mesenchymal stem cells (BMSCs, CRL-12424, ATCC, Manassas, VA, USA), which were tested to be free from mycoplasma contamination, were cultured until achieving ~ 75% confluence, and the BMSCs, before passage 5, were used for an all cell assay. BMSCs were trypsinized and seeded in 24-well plates in normal growth media (10% fetal bovine serum+Dulbecco’s modified Eagle’s medium, Gibco) at a density of 5000 cells per cm^2^ for morphology observation and at 10 000 cells per cm^2^ for the osteogenesis experiments. The samples with the seeded cells were placed in a 37 °C incubator under a 5% CO_2_ atmosphere.

### BMSC morphology and proliferation

To observe the cell morphology, BMSCs were cultured on samples for 2 days and fixed in 4% paraformaldehyde for 10 min. The cells were then stained with an Actin-Tracker Green (Beyotime, Beijing, China) solution that contained 1% bovine serum albumin and 0.1% Triton X-100. Subsequently, cells were also stained with 4′,6-diamidino-2-phenylindole (DAPI; Sigma, St Louis, MO, USA). Images of the cells were observed using a confocal laser scanning microscope (LSM700, Carl Zeiss Group). Before observation, the boarder of the NT and IT were fixed perpendicular to the x axis of the microscope direction. A CCK-8 (Dojindo, Kumamoto, Japan) assay was used to evaluate the proliferation rate of cells growing on the surface of the samples. After incubation for 1, 3 and 5 days, the culture medium was aspirated, and an aliquot of CCK-8 (10%, 100 μl) dissolved in Dulbecco’s modified Eagle’s medium was added to each sample. The plates were then incubated at 37 °C for 2 h. The CCK-8-containing solution was transferred to a 96-well plate, and the absorbance at 450 nm was measured using a spectral scanning multimode reader (Varioskan Flash, Thermo-Fisher Scientific, Winsford, UK). A live/dead staining assay was carried out on the BMSCs cultured on the MEFs. The BMSCs were seeded on the MEF surface at a density of 2 × 10^4^ cells per cm^2^ and cultured for 24 h. Then, the cells were rinsed with phosphate buffer solution and incubated in a mixture of 2μg ml^−1^ calcein acetomethoxy and 5 μg ml^−1^ propidium iodide for 15 min. Fluorescent images of the cells were obtained using a fluorescence microscope (Eclipsc Ti-U, Nikon, Tokyo, Japan).

### ALP activity and detection of mineralized product

Alkaline phosphatase (ALP) activity was measured by quantifying the amount of the p-nitrophenol converted from p-nitrophenol phosphate using a microplate absorbance reader at 405 nm and then dividing by the cell protein concentration. After 7 days, the cells were lysed in 0.2% Triton solution for examination. For Alizarin Red S staining, cells cultured for 21 days were rinsed in phosphate buffer solution and fixed with 10% formalin. The fixed cells were stained in 2% (wt/v) Alizarin Red S solution (pH =4.2). After incubation for 10 min, the Alizarin Red S solution was aspirated, and the cells were washed with distillated water. The stained samples were observed using a three-dimensional light microscope (HiroX7700, Hirox Co., Ltd., Tokyo, Japan).

### *In vivo* evaluation of MEF

To evaluate the MEF-induced bone regeneration *in vivo*, we used a rat femoral defect model. All animal studies were approved by the animal care and use committee of General Hospital of Guangzhou Military Command of PLA. To acquire the rat femoral defect model, Sprague Dawley rats (male, 12 weeks old) were used. The animals were randomized into five groups: control 1 (pure Ti), control 2 (pristine NT TiO_2_, the substrate without any laser interaction), control 3 (substrate with the full surface being scanned by laser), 30-MEF and 75-MEF. The investigators were not blinded to the group assignments. The number of animals in each group was chosen to be 6 according to a power analysis. The animals were first anesthetized with chloral hydrate, and then a femoral defect was created at the intercondylar notch using a low rotation speed surgical motor. The MEF specimens and their controls (~10 mm long, 2 mm wide) were implanted into the defect sites. The animals were killed after 6 weeks, and bone samples were analyzed using micro-computed tomography (Latheta, Aloka co., LTD, Tokyo, Japan) and histological analysis. The non-decalcified bones with implanted specimens were dehydrated in graded ethanol. Then, these specimens were immersed into a mixture of the methyl methacrylate monomer, dibutyl phthalate and perkadox to initiate polymerization. After polymerization, the specimens embedded in polymethyl methacrylate were cut into sections in the direction vertical to the long axis of the implants using cutting equipment (SP1600, Leica, Solms, Germany), and then the specimen sections were ground to a thickness of ~ 50 μm using grinding equipment (EXAKT-400, Exakt, Norderstedt, Germany). The sections were stained light green and observed using a light microscope (Axioskop 40 POL, Carl Zeiss Group).

### Statistical analysis

Data are presented as the mean ± s.d. Significant differences (*P<0.05, **P<0.01, *N* =6) in the data were analyzed using SPSS (IBM, Armonk, NY, USA) with a one-way analysis of variance.

## RESULTS AND DISCUSSION

**Figure 2:**
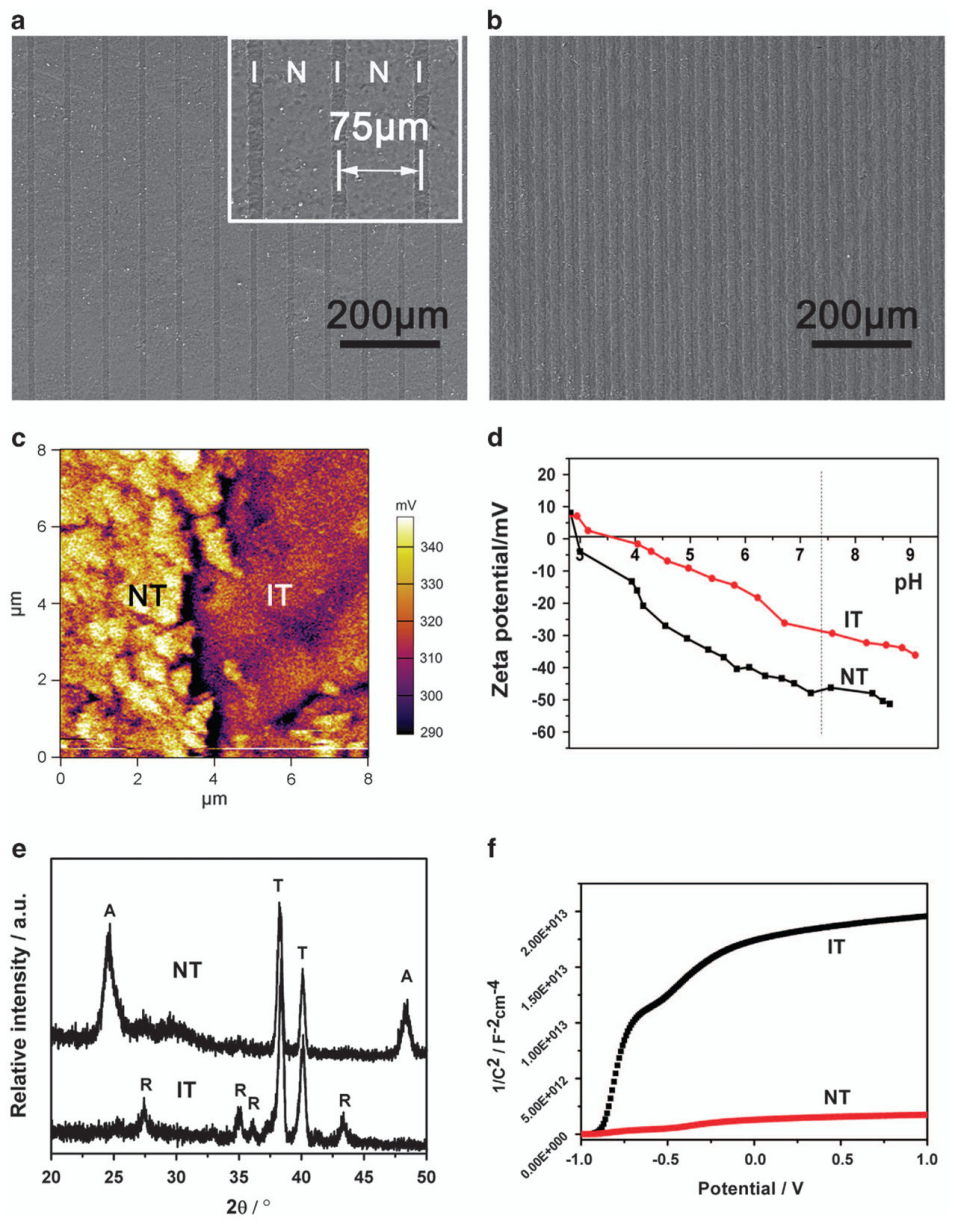
Construction of the microscale electrostatic field (MEFs). (**a**, **b**) Scanning electron microscopy (SEM) images of constructed MEFs with different charged domain intervals ((**a**) 75 μm interval, (**b**) 30 μm interval) on the titanium surface. N refers to NT (non-irradiated n-type semiconducting anatase TiO_2_ zone prepared by hydrothermal synthesis), and I refers to IT (n-type semiconducting rutile TiO_2_ zone prepared by laser irradiation of the anatase TiO_2_ zone). The inset is the magnified view of the SEM image. (**c**) Kelvin probe force microscopy (KPFM) image of the border of the NT (left) and IT zones (right) of the MEF sample with a 30 μm interval showing that the relative potential on the NT zone is ~ 19 mV greater than that on the IT zone. (**d**) The zeta potential analysis of the MEF sample suggests that the zeta potential around a pH of 7.4 for NT is less than that for IT. (**e**) X-ray diffraction (XRD) patterns of NT and IT indicate the NT and IT are mainly composed of anatase and rutile, respectively. A, anatase; R, rutile; T, titanium. (**f**) Mott–Schottky plots for the NT and IT surface indicating they are both n-type semiconductors, and their carrier density (N_D_) values were 1.82 × 10^19^ cm ^−3^ and 9.18 × 10^20^ cm ^−3^, respectively.

We utilized selective laser irradiation to site specifically convert some areas of a uniform hydrothermally formed n-type semiconducting anatase phase film into n-type semiconducting rutile phase. Consequently, the NT and IT zones are periodically organized on the implant surface. Specifically, a pristine TiO_2_ film was first prepared on the titanium surface by hydrothermal synthesis followed by heat treatment. Computer-controlled laser irradiation was carried out on pristine TiO_2_ to construct parallel IT stripes on the titanium surface. During the laser irradiation process, the focused laser beam selectively irradiated the confined area of the pristine TiO_2_. Under the proper laser power, the focused beam could cause an increase in the temperature in the confined microscale area.^24,25^ Finally, a periodic distribution of microscale NT and IT zones could be achieved on the titanium surface. The intervals between the NT and IT zones could be tuned by simply designing the laser scanning distance using a computer program. Here, the implants modified with a laser scanning interval of 75 and 30 μm were referred to as 75-MEF and 30-MEF, respectively (Figure 2a and b). Because the laser irradiation zone is 13.6 ± 0.5 μm wide when a 10 μm laser scanning distance was applied, the NT film would be completely irradiated, generating a film completely covered by IT.

The surface potential and the compositional analysis of the NT and IT zones were performed to confirm the construction of the MEF. The surface potential was examined by using Kelvin probe force microscopy (carried out on 30-MEF) to determine the periodic surface potential difference between the NT and IT zones. Kelvin force microscopy analysis indicated that the surface potential of the IT zone was ~ 19 mV negatively shifted compared with that of the NT zone (Figure 2c). For further confirmation of the surface potential distribution, Kelvin force microscopy images of 8 successive domains on the 30-MEF sample were obtained. These images indicated that the domains (NT and IT) with different surface potentials were periodically distributed on the titanium surface (Supplementary Figure S1). Further charge examination was performed by analyzing the zeta potential of the NT and IT films. The measurement identified the difference in the zeta potential before and after laser irradiation with the IT showing a greater zeta potential at pH ~ 7.4 (Figure 2d). The fact that in-solution NT films are more negatively charged is consistent with the Kelvin force microscopy results. This result indicates that when NT and IT are distributed as zones on a film, there are differences in the surface charge between the NT and IT zones, generating a MEF on the implant surface.

Energy-dispersive X-ray spectroscopic line scanning confirms that there is no change in elemental composition across the border between the NT and IT zones (Supplementary Figure S2). The phase compositions of the NT and IT films were examined using the X-ray diffraction spectra in Figure 2e, where A, R and T refer to anatase, rutile and titanium, respectively. The results obviously show that the NT and IT are mainly composed of an anatase and rutile phase, respectively.^26,27^ Mott–Schottky analysis is frequently applied to investigate the semiconductor/electrolyte interface.^28,29^ The carrier density could be obtained from the slope of Mott–Schottky plots using Equation (1)

(1) $$C^{-2}=\frac{2(E-E_{fb}-kT/e)}{N_{D}\varepsilon \varepsilon_{0}eA^{2}}$$

where *N*_D_ is the electron density, *C* is the space charge capacitance, *E*_fb_ is flat band potential and *A* is the active surface. The electron density of the electrode can be calculated from the intercept and the slope. Here, *ε* is the dielectric constant of TiO_2_, *ε*_0_ is the vacuum permittivity, *k* is the Boltzmann constant, *T* is the absolute temperature and *e* is the elementary charge.

As shown in Figure 2f, both NT and IT films exhibit a positive slope in the Mott – Schottky plots indicating they are both n-type semiconductors.^29,30^ Electron carrier densities of NT and IT samples were calculated to be 1.82 × 10^19^ cm^−3^ and 9.18 × 10^20^ cm^−3^, respectively. After laser irradiation, the carrier density of the IT zone was increased because of the phase transition and defect formed during laser irradiation.^21,31,32^ Based on the observations outlined above, we conclude that a MEF was constructed on the titanium surface, as shown in Figure 1.

**Figure 3:**
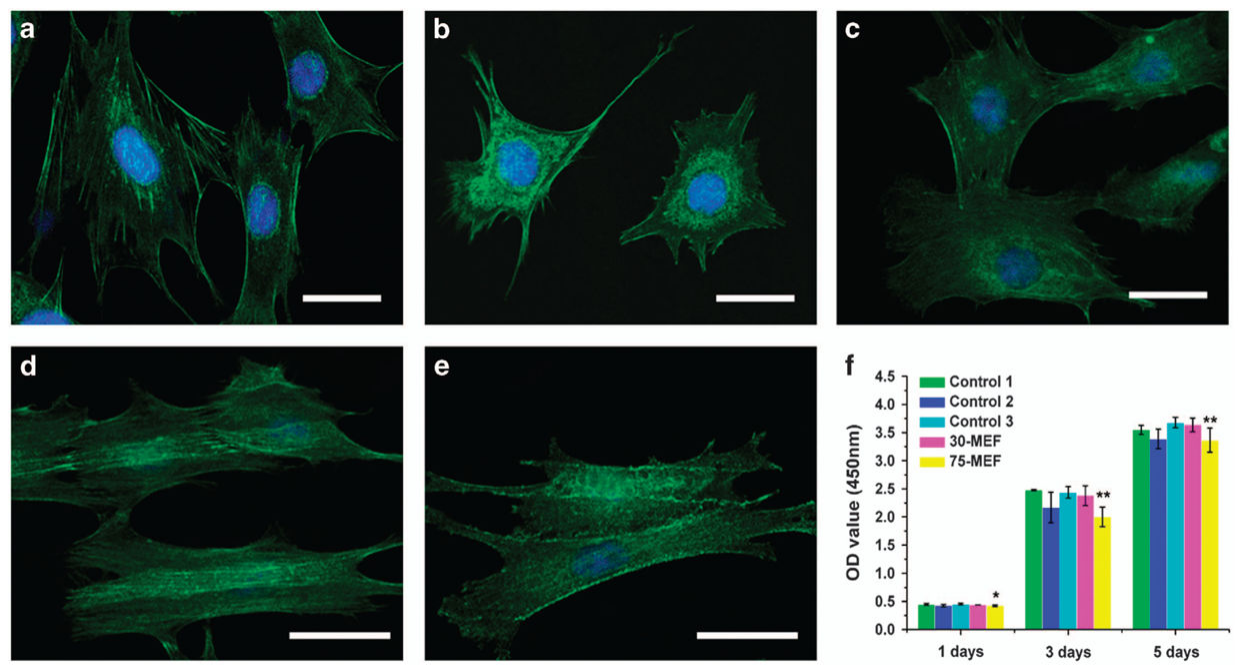
Morphology and proliferation of bone marrow-derived mesenchymal stem cells (BMSCs) on the microscale electrostatic field (MEF) surface and control samples. (**a**–**e**) Fluorescence images of the BMSC skeletons on the MEF surface and the control samples after being cultured for 2 days: (**a**) control 1 (pure Ti), (**b**) control 2 (pristine NT TiO_2_, the substrate without any laser interaction), (**c**) control 3 (substrate with the full surface being scanned using the laser), (**d**) 30-MEF and (**e**) 75-MEF. These images show that the BMSCs on 30-MEF are elongated and aligned along the MEF substrate. Cell nuclei were stained by 4′,6-diamidino-2-phenylindole (DAPI; blue), and F-actin was stained by fluorescein isothiocyanate (FITC)-labeled phalloidin (green). (**f**) CCK-8 assay of the MEF surfaces showing that the 75-MEF prohibits cell proliferation. Scale bar: 50 μm. Values represent the mean ± s.d.. *Significant difference at *P*<0.05; **significant difference at *P*<0.01.

To determine whether cells cultured on MEFs could induce and promote osteogenic differentiation and bone regeneration, we prepared three control groups without MEFs. The controls included etched pure titanium (control 1), a pristine NT film prepared by hydrothermal synthesis (control 2) and an IT film prepared by laser irradiation of the whole pristine TiO_2_ film (control 3). Rat BMSCs were cultured on these substrates to evaluate the cellular behavior under the guidance of MEFs. At first, the cell morphology was examined by immunofluorescent staining of the actin cytoskeleton using phalloidin. BMSCs on all three control samples were found to have a spreading morphology, with control 2 showing less spreading area. MEFs were also found to induce elongation and parallel alignment of the BMSCs along the direction perpendicular to the border of the NT and IT zones (Figure 3). When a cell is cultured in the presence of the MEF, there is a gradient of charge across the insulating plasma between the cell membranes. During this process, MEF influenced the ion channels and the electrophoresis of membrane proteins, resulting in the regulation of cytoskeletal elements and cell morphology.^6^ The cell morphology and organization and orientation of cytoskeleton would have an effect on the subsequent stem cell differentiation.^33^ A CCK-8 assay was conducted to further examine the BMSC proliferation ability on MEFs. This assay showed that the pristine NT TiO_2_ sample (control 3) and the 30-MEF sample had similar cell viabilities to control 1 and control 2. The cell proliferation ability was slightly depressed on the 75-MEF (Figure 3f). Meanwhile, a live/dead assay was carried out to test the BMSC cytotoxicity of the MEFs. The live cells were stained green, and the dead cells were stained red. The results in Supplementary Figure S3 indicate that almost all BMSCs were alive after being cultured on the MEFs for 24 h. Therefore, it can be concluded that the MEF samples were not toxic to the cells.

**Figure 4:**
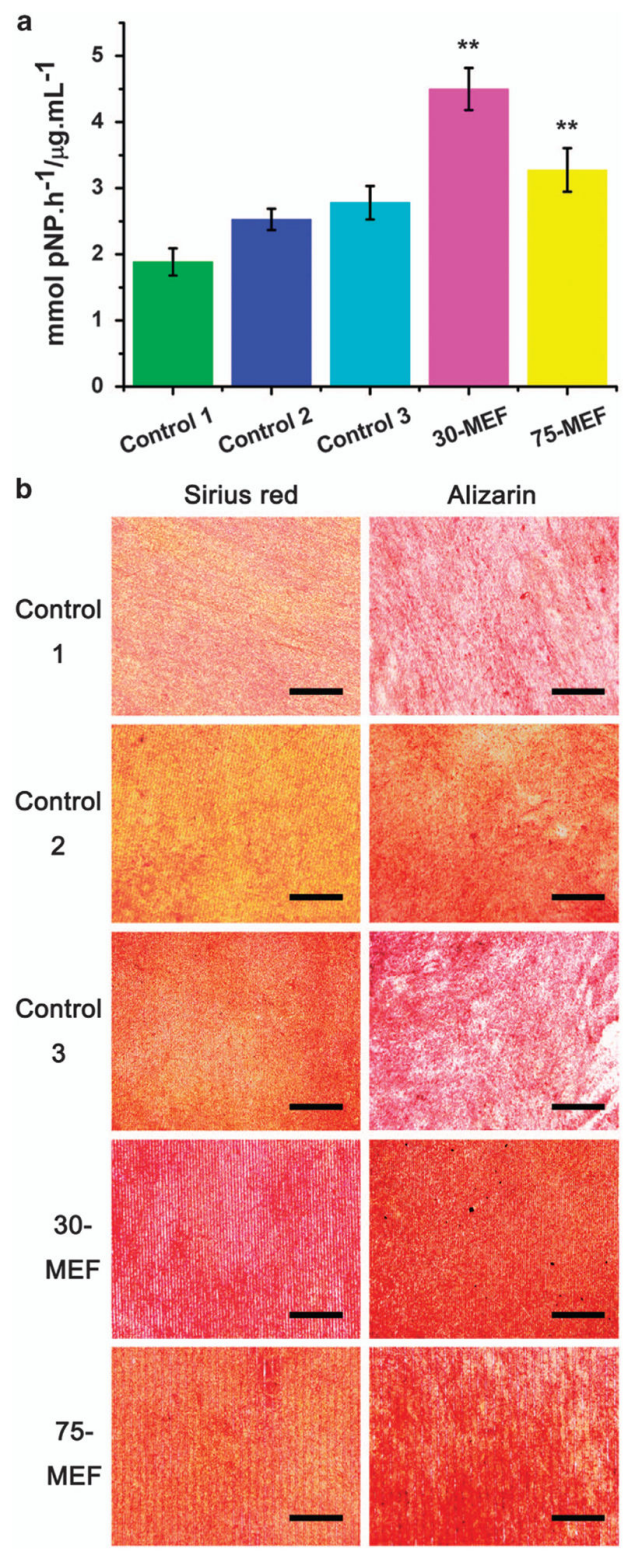
Effect of the microscale electrostatic field (MEF) on the osteogenic differentiation of bone marrow-derived mesenchymal stem cells (BMSCs). (**a**) Alkaline phosphatase (ALP) enzymatic activity of the BMSCs cultured on the control groups and MEF samples in the growth media showing that the MEF with a 30 μm interval upregulates ALP activity. The samples in each experiment were performed in quadruplicate, and the experiment was repeated twice (**P<0.01). (**b**) Osteogenic differentiation visualized using Sirius Red staining and Alizarin Red staining after 14 and 21 days of incubation in growth media, respectively, suggesting that the 30-MEF induces collagen secretion and the subsequent calcium mineralization. These results indicate that the MEF with a 30-μm interval most significantly induces and promotes osteogenesis. Scale bar: 500 μm.

To identify the effect of the MEFs on BMSC differentiation, the ALP activity and the osteogenic products were examined. ALP is the major osteogenic marker during the early time points of osteogenesis. The cells were cultured in growth media (that is, non-osteogenic media) to exclude the influence of osteogenic inducers in the media.^34,35^ On day 7, both the 30-MEF and the 75-MEF significantly enhanced the expression of ALP, indicating that the MEF could induce BMSCs to achieve osteogenesis in the absence of osteogenic supplements. In addition, on day 7, an almost twofold increase in ALP activity could be observed on 30-MEF compared with the controls, suggesting that 30-MEF has the greatest osteogenesis-inducing capability among all samples studied, including the controls without a MEF (Figure 4a). The 75-MEF samples also showed greater ALP activities compared with the controls. Controls 1, 2 or 3 did not effectively promote osteogenesis because of the absence of osteogenic inducers in the media and their own incapability of inducing osteogenic differentiation. The promotion of increased mineralization on the MEFs indicates that MEFs with adequate intervals could promote BMSC osteogenesis.^36,37^ To confirm and analyze the mineralization process of the MEFs, the cells on days 14 and 21 were stained using Sirius Red and Alizarin Red to confirm the formation of collagen and calcium nodules, respectively (Figure 4b). At the beginning of biomineralization, differentiated osteoblasts secreted collagen, the precursor for bone mineralization.^38^ More secreted collagen could be observed on the 30-MEFs (stained in red). The Alizarin Red staining further confirmed the formation of abundant calcium nodules on the 30-MEFs. These results confirmed that the 30-MEF could induce BMSC differentiation and produce mineralized particles most efficiently.

Various studies have confirmed that externally added electric fields can modulate cell differentiation.^4,5,15,16^ In this study, the electric field was not externally applied but was generated and built-in because of the unique periodic organization of the two n-type semiconducting zones, NT and IT, that have different electron carrier densities (Figure 1). When BMSCs were cultured on the MEF, the cells would respond to the built-in MEF. The response is mainly induced because of the presence of charged molecules, such as the transmembrane proteins, ion channel proteins and phosphatidylserine on the surface of the cell membrane.^8,9,11^ The MEF would serve as an effective electrical cue for guiding cell differentiation. We believe that the MEF is comparable to the built-in endogenous electric field in natural bone because of the piezoelectric nature of bone collagen.^1,2^

**Figure 5:**
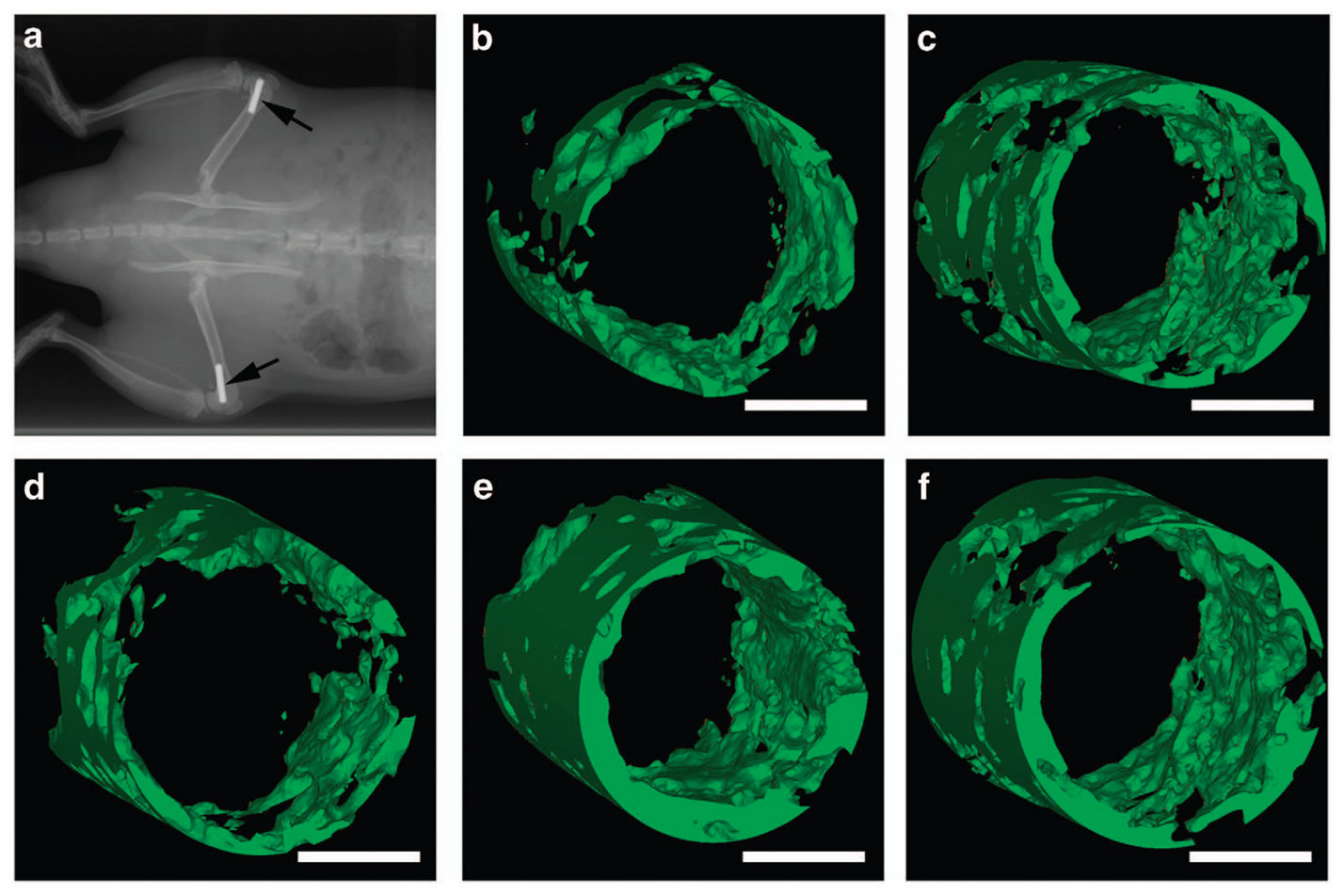
*In vivo* osteogenesis on the cylindrical Ti implants with the microscale electrostatic field (MEF) on the surfaces after 6 weeks. (**a**) X-ray image of the rat showing the implantation site in the rat femur. The black arrows highlight the position of the implantation. (**b–f**) Three-dimensional (3D) reconstructed micro-computed tomography (micro-CT) images of the new bone tissue formed around the implanted Ti cylinders with the MEF on the surfaces ((**b**) control 1, (**c**) control 2, (**d**) control 3, (**e**) 30-MEF and (**f**) 75-MEF). The quantitative results suggest that the new bone tissue formed around the implanted control 1, control 2, control 3, 30-MEF and 75-MEF Ti cylinders were 2.00, 3.38, 2.16, 5.29 and 3.80 mm^3^, respectively, suggesting that the 30-MEF sample most efficiently induced and promoted bone regeneration compared with the other groups. Scale bars: 1 mm.

To evaluate whether the MEF could induce bone formation *in vivo*, the 75-MEF, 30-MEF and the three controls were also fabricated on cylindrical titanium rods (2 mm in diameter) using a laser scanner controlled with a step motor. The samples were implanted into a rat femoral condyle.^39^ Figure 5a illustrates that the sample was implanted into the distal femoral condyles of Sprague Dawley rats. At 6 weeks after implantation, the femoral bones with implants were embedded in methyl methacrylate polymer, sectioned and ground to reach a thickness of ~50 μm. The samples were also analyzed using micro-computed tomography. Three-dimensional reconstructions of the micro-computed tomography images are shown in Figure 5b–f. The quantitative analysis of the three-dimensional reconstructed images suggests that the new bone tissue formed around the implanted control 1, control 2, control 3, 30-MEF and 75-MEF Ti cylinders was 2.00, 3.38, 2.16, 5.29 and 3.80 mm^3^, respectively. These results confirmed that the 30-MEF samples could significantly induce bone regeneration to achieve bone repair compared with the other groups. The differentiation of stem cells *in vivo* was mediated by the electrical cues provided by the surrounding environment.^10,40^ The electrical stimulus was transformed into the endogenous electric field that carried information to guide BMSC differentiation.^40^ Therefore, it is possible that the built-in MEF on the implants guided bone regeneration.

Histological sections of the bone-implant constructs were stained light green to examine the bone–implant interface.^41^ The newly formed bone (trabeculae) was stained dark green. Generally, only a small percentage of newly formed bone was in direct contact with the control implant samples (Supplementary Figure 4a–c), which is a signature of poor osteoinduction and integration around the control samples. However, for the MEF implants, the amount of newly formed bones in direct contact with the implant surface was greatly increased. Moreover, more newly formed bone in contact with implants was found on the 30-MEF than on the 75-MEF. These results further confirm that the implants with 30-MEF could induce and promote bone regeneration most efficiently.

## CONCLUSION

A periodic MEF was built-in to bone implants to serve as a novel electrical cue for directing and inducing osteogenic differentiation and bone regeneration. The periodic MEF was generated as a result of the periodic distribution of two n-type semiconducting TiO_2_ phases, anatase and rutile, and were made to have lower and higher electron densities, respectively. The periodic distribution of the anatase and rutile zones was achieved using selective laser irradiation. The built-in MEF was found to induce the osteogenic differentiation of the BMSCs even in the absence of osteogenic supplements, and this was demonstrated by the fact that the stem cells showed enhanced ALP activity and increased osteogenic products on the implants with the built-in MEFs compared with the control samples. *In vivo* implantation further confirmed that the MEF could significantly induce and promote bone regeneration around the bone implants. Because the MEF was built-in but not externally applied to the implant surface, the use of a MEF is a convenient and safe approach to inducing osteogenesis and bone formation around implants to achieve excellent osteointegration. In essence, our work opens up a new avenue for bone repair and regeneration.

## References

[1] Shamos MH, Lavine LS, Shamos MI (1963). Piezoelectric effect in bone. Nature.

[2] Minary-Jolandan M, Yu MF (2009). Uncovering nanoscale electromechanical heterogeneity in the subfibrillar structure of collagen fibrils responsible for the piezoelectricity of bone. ACS Nano.

[3] Voldman J (2006). Electrical forces for microscale cell manipulation. Annu Rev Biomed Eng.

[4] Cui H, Wang Y, Cui L, Zhang P, Wang X, Wei Y, Chen X (2014). *In vitro* studies on regulation of osteogenic activities by electrical stimulus on biodegradable electroactive polyelectrolyte multilayers. Biomacromolecules.

[5] Hess R, Jaeschke A, Neubert H, Hintze V, Moeller S, Schnabelrauch M, Wiesmann HP, Hart DA, Scharnweber D (2012). Synergistic effect of defined artificial extracellular matrices and pulsed electric fields on osteogenic differentiation of human MSCs. Biomaterials.

[6] Campetelli A, Bonazzi D, Minc N (2012). Electrochemical regulation of cell polarity and the cytoskeleton. Cytoskeleton.

[7] Liao J, Zhu Y, Zhou Z, Chen J, Tan G, Ning C, Mao C (2014). Reversibly controlling preferential protein adsorption on bone implants by using an applied weak potential as a switch. Angew Chem Int Ed.

[8] Abed E, Martineau C, Moreau R (2011). Role of melastatin transient receptor potential 7 channels in the osteoblastic differentiation of murine MC3T3 cells. Calcified Tissue Int.

[9] Yeung T, Gilbert GE, Shi J, Silvius J, Kapus A, Grinstein S (2008). Membrane phosphatidylserine regulates surface charge and protein localization. Science.

[10] Allen GM, Mogilner A, Theriot JA (2013). Electrophoresis of cellular membrane components creates the directional cue guiding keratocyte galvanotaxis. Curr Biol.

[11] Pfreundschuh M, Hensen U, Muller DJ (2013). Quantitative imaging of the electrostatic field and potential generated by a transmembrane protein pore at subnanometer resolution. Nano Lett.

[12] Alexander RT, Jaumouillé V, Yeung T, Furuya W, Peltekova I, Boucher A, Zasloff M, Orlowski J, Grinstein S (2011). Membrane surface charge dictates the structure and function of the epithelial Na^+^/H^+^ exchanger. EMBO J.

[13] Hu WW, Hsu YT, Cheng YC, Li C, Ruaan RC, Chien CC, Chung CA, Tsao CW (2014). Electrical stimulation to promote osteogenesis using conductive polypyrrole films. Mater Sci Eng C.

[14] Hardy JG, Villancio-Wolter MK, Sukhavasi RC, Mouser DJ, Aguilar D, Geissler SA, Kaplan DL, Schmidt CE (2015). Electrical stimulation of human mesenchymal stem cells on conductive nanofibers enhances their differentiation toward osteogenic outcomes. Macromol Rapid Comm.

[15] McCullen SD, McQuilling JP, Grossfeld RM, Lubischer JL, Clarke LI, Loboa EG (2010). Application of low-frequency alternating current electric fields via interdigitated electrodes: effects on cellular viability, cytoplasmic calcium, and osteogenic differentiation of human adipose-derived stem cells. Tissue Eng Part C.

[16] Zou H, Mellon S, Syms RRA, Tanner KE (2006). 2-dimensional MEMS dielectrophoresis device for osteoblast cell stimulation. Biomed Microdevices.

[17] Hammerick KE, Longaker MT, Prinz FB (2010). In vitro effects of direct current electric fields on adipose-derived stromal cells. Biochem Biophs Res Comm.

[18] Nair AK, Gautieri A, Chang SW, Buehler MJ (2013). Molecular mechanics of mineralized collagen fibrils in bone. Nat Commun.

[19] Rigby SJ, Al-Obaidi AHR, Lee SK, McStay D, Robertson PKJ (2006). The application of Raman and anti-stokes Raman spectroscopy for in situ monitoring of structural changes in laser irradiated titanium dioxide materials. Appl Surf Sci.

[20] Lee SK, Robertson PKJ, Mills A, McStay D, Elliott N, McPhail D (2003). The alteration of the structural properties and photocatalytic activity of TiO_2_ following exposure to non-linear irradiation sources. Appl Catal B.

[21] Scanlon DO, Dunnill CW, Buckeridge J, Shevlin SA, Logsdail AJ, Woodley SM, Catlow CR, Powell MJ, Palgrave RG, Parkin IP, Watson GW, Keal TW, Sherwood P, Walsh A, Sokol AA (2013). Band alignment of rutile and anatase TiO_2_. Nat Mater.

[22] Liu L, Chen X (2014). Titanium dioxide nanomaterials: self-structural modifications. Chem Rev.

[23] Nel AE, Mädler L, Velegol D, Xia T, Hoek EMV, Somasundaran P, Klaessig F, Castranova V, Thompson M (2009). Understanding biophysicochemical interactions at the nano-bio interface. Nat Mater.

[24] Lu JP, Lim X, Zheng MR, Mhaisalkar SG, Sow CH (2012). Direct laser pruning of CdS_x_Se_1−x_ nanobelts en route to a multicolored pattern with controlled functionalities. ACS Nano.

[25] Mohanta RR, Medicherla VRR, Mohanta KL, Nayak NC, Majumder S, Solanki V, Varma S, Bapna K, Phase DM, Sathe V (2015). Ion beam induced chemical and morphological changes in TiO_2_ films deposited on Si(111) surface by pulsed laser deposition. Appl Surf Sci.

[26] Li JG, Ishigaki T, Sun XD (2007). Anatase, brookite, and rutile nanocrystals via redox reactions under mild hydrothermal conditions: phase-selective synthesis and physicochemical properties. J Phys Chem C.

[27] Koparde VN, Cummings PT (2008). Phase transformations during sintering of titania nanoparticles. ACS Nano.

[28] Wang MY, Ioccozia J, Sun L, Lin CJ, Lin ZQ (2014). Inorganic-modified semiconductor TiO2 nanotube arrays for photocatalysis. Energ Environ Sci.

[29] Tay QL, Liu X, Tang Y, Jiang Z, Sum TC, Chen Z (2013). Enhanced photocatalytic hydrogen production with synergistic two-phase anatase/brookite TiO_2_ nanostructures. J Phys Chem C.

[30] Wolcott A, Smith WA, Kuykendall TR, Zhao YP, Zhang JZ (2009). Photoelectrochemical water splitting using dense and aligned TiO_2_ nanorod arrays. Small.

[31] Huang CN, Bow JS, Zheng Y, Chen SY, Ho N, Shen P (2010). Nonstoichiometric titanium oxides via pulsed laser ablation in water. Nanoscale Res Lett.

[32] Apgar BA, Lee SK, Schroeder LE, Martin LW (2013). Enhanced photoelectrochemical activity in all-oxide heterojunction devices based on correlated “metallic” oxides. Adv Mater.

[33] Watt FM, Huck WTS (2013). Role of the extracellular matrix in regulating stem cell fate. Nat Rev Mol Cell Bio.

[34] Gaharwar AK, Mihaila SM, Swami A, Patel A, Sant S, Reis RL, Marques AP, Gomes ME, Khademhosseini A (2013). Bioactive silicate nanoplatelets for osteogenic differentiation of human mesenchymal stem cells. Adv Mater.

[35] Yao X, Peng R, Ding JD (2013). Effects of aspect ratios of stem cells on lineage commitments with and without induction media. Biomaterials.

[36] Neufurth M, Wang X, Schröder HC, Feng Q, Diehl-Seifert B, Ziebart T, Steffen R, Wang S, Müller WE (2014). Engineering a morphogenetically active hydrogel for bioprinting of bioartificial tissue derived from human osteoblast-like SaOS-2 cells. Biomaterials.

[37] Shimizu M, Kobayashi Y, Mizoguchi T, Nakamura H, Kawahara I, Narita N, Usui Y, Aoki K, Hara K, Haniu H, Ogihara N, Ishigaki N, Nakamura K, Kato H, Kawakubo M, Dohi Y, Taruta S, Kim YA, Endo M, Ozawa H, Udagawa N, Takahashi N, Saito N (2012). Carbon nanotubes induce bone calcification by bidirectional interaction with osteoblasts. Adv Mater.

[38] Palmer LC, Newcomb CJ, Kaltz SR, Spoerke ED, Stupp SI (2008). Biomimetic systems for hydroxyapatite mineralization inspired by bone and enamel. Chem Rev.

[39] Alghamdi HS, Bosco R, van den Beucken JJJP, Walboomers XF, Jansen JA (2013). Osteogenicity of titanium implants coated with calcium phosphate or collagen type-I in osteoporotic rats. Biomaterials.

[40] Levin M (2013). Reprogramming cells and tissue patterning via bioelectrical pathways: molecular mechanisms and biomedical opportunities. Wires Syst Biol Med.

[41] Li HS, Zou X, Xue Q, Egund N, Lind M, Bünger C (2004). Anterior lumbar interbody fusion with carbon fiber cage loaded with bioceramics and platelet-rich plasma: an experimental study on pigs. Eur Spine J.

[42] Yuan YP, Ruan LW, Barber J, Loo SCJ, Xue C (2014). Hetero-nanostructured suspended photocatalysts for solar-to-fuel conversion. Energ Environ Sci.

